# Cities and armed conflict: A systematic urban-rural coding of UCDP conflict events data

**DOI:** 10.1016/j.dib.2021.107554

**Published:** 2021-11-07

**Authors:** Emma Elfversson

**Affiliations:** Department of Peace and Conflict Research, Uppsala University, Box 514, Uppsala 751 20, Sweden

**Keywords:** Cities, Armed conflict, Urban violence, Conflict events, Political violence

## Abstract

This data article provides a descriptive overview of the Cities and Armed Conflict Events (CACE) dataset and the data collection methods. The dataset provides a systematic coding of armed conflict events taking place in cities and outside cities across the globe. It constitutes an extension of the Uppsala Conflict Data Program (UCDP) Georeferenced Events Dataset (GED) version 18.1 and covers 1989–2017. To identify which events of armed conflict took place in cities, the data was manually matched to to data from the United Nations Statistics Division (UNSD). The dataset enables systematic analysis of urban-rural patterns in armed conflict, as illustrated by Elfversson & Höglund [1]. While existing methods for analysing such patterns frequently rely on matching conflict data to spatial grids combined with population density, the data presented here with higher validity captures whether violent events take place in cities.

## Specifications Table

**Table:** 

Subject	Political Science
Specific subject area	Peace and conflict research
Type of data	Table
How data were acquired	The data on conflict events is from the Uppsala Conflict Data Program which relies on news articles, complemented by reports from NGOs and other organizations as well as academic studies. To extend this dataset and code for each event whether it occurred in a city, we primarily rely on information from the United Nations Statistics Division which in turn relies on official demographic data from national statistical offices. The data was compiled, and descriptive analysis conducted, in Excel.
Data format	Raw
Parameters for data collection	All events contained in the UCDP GED (v 18.1) were included. The dataset covers the period 1989–2017, and is global in scope, except for Syria which is not included in UCDP GED v 18.1.
Description of data collection	From the UNSD cities data, all cities in countries with UCDP GED events were extracted, making up a total list of 3956 cities. The operational threshold for inclusion was that the city must have a population of at least 100 000 at some point since 1989. After establishing the list of cities, all the events contained in the UCDP GED were coded according to whether or not they occurred in one of the cities on our list, based on the location description in the dataset (“where”). This matching was conducted manually, taking into account different spellings of city names and, when necessary, double-checking the match using the georeferences provided in the UCDP GED dataset.
Data source location	Primary data sources: Uppsala Conflict Data Program Institution: Uppsala University City/Town/Region: Uppsala Country: Sweden United Nations Statistics Division Institution: United Nations City/Town/Region: New York, N.Y. Country: United States
Data accessibility	The dataset with the accompanying codebook is deposited on GitHub, where it is freely available to download. Direct URL to data: https://github.com/emmaelfversson/cace. DOI: 10.5281/zenodo.5635672 The data, and possible future updates, are also available at the Uppsala Conflict Data Program's data repository: https://ucdp.uu.se/downloads/
Related research article	E. Elfversson & K. Höglund, Are armed conflicts becoming more urban? *Cities* 119 (2021). https://doi.org/10.1016/j.cities.2021.103356

## Value of the Data

• The dataset provides an important extension of an existing world-leading conflict events dataset by systematically coding which events take place in cities. This new category of information enables studies of urban/rural patterns in violence that rely on a more valid measure of cities than approaches based on spatial grids combined with population density.
• The data will be useful for studies on spatial conflict dynamics, particularly for studies that focus on cities and urban dynamics as a unit of analysis or predictor.
• For example, the data may be used to study under what conditions conflicts are likely to affect cities, if conflicts become more or less urban over time, and if different forms of organized violence affect cities differently.

## 1 Data Description

The dataset “Cities and armed conflict events” consists of two data files (the main events dataset, provided in Excel and csv format, and a list of cities) and a Codebook which provides information on the variables contained in the data file. The dataset constitutes an extension of the Uppsala Conflict Data Program (UCDP) Georeferenced Events Dataset (GED) [2] which is a widely used dataset for studies of organized violence and armed conflict. The UCDP GED systematically codes events of violence for armed conflicts which result in at least 25 battle-related deaths (see further Sundberg & Melander [2]). The Cities and Armed Conflict Events (CACE) dataset provides a systematic coding of whether these armed conflict events took place in cities. To identify which events of armed conflict took place in cities, the data was manually matched with data from the United Nations Statistics Division (UNSD).

**Fig. 1:**
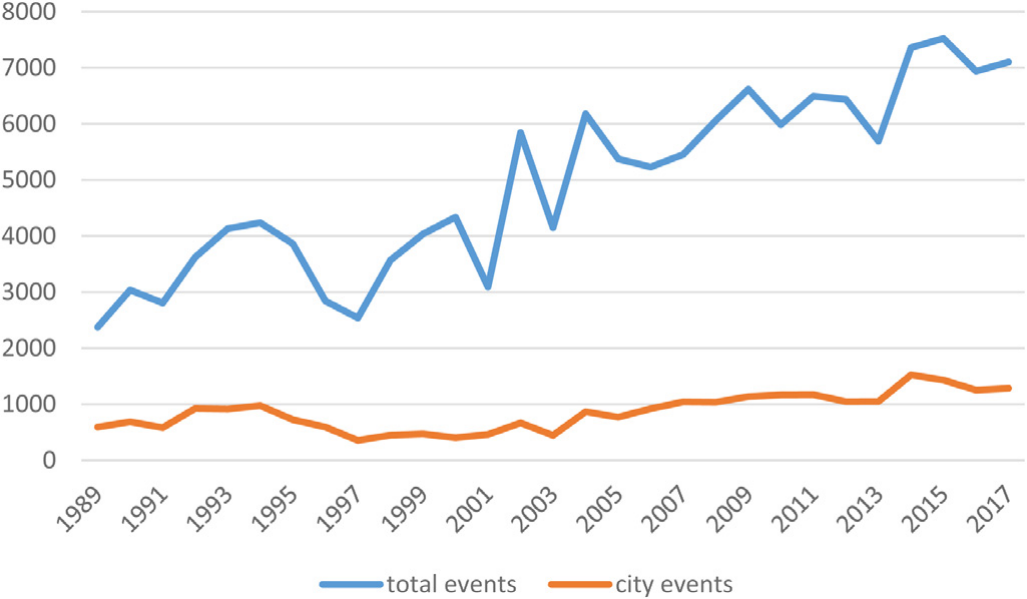
Patterns of violence (events).

In total, the dataset contains 142 902 conflict events. According to CACE, 24 904 of the armed conflict events (17%), and 294 158 (15%) of the total fatalities, took place in cities. Fig. 1 below illustrates the pattern over time in terms of events. The blue line denotes the total number of events each year, whereas the red line denotes the number of events that took place in cities.

**Fig. 2:**
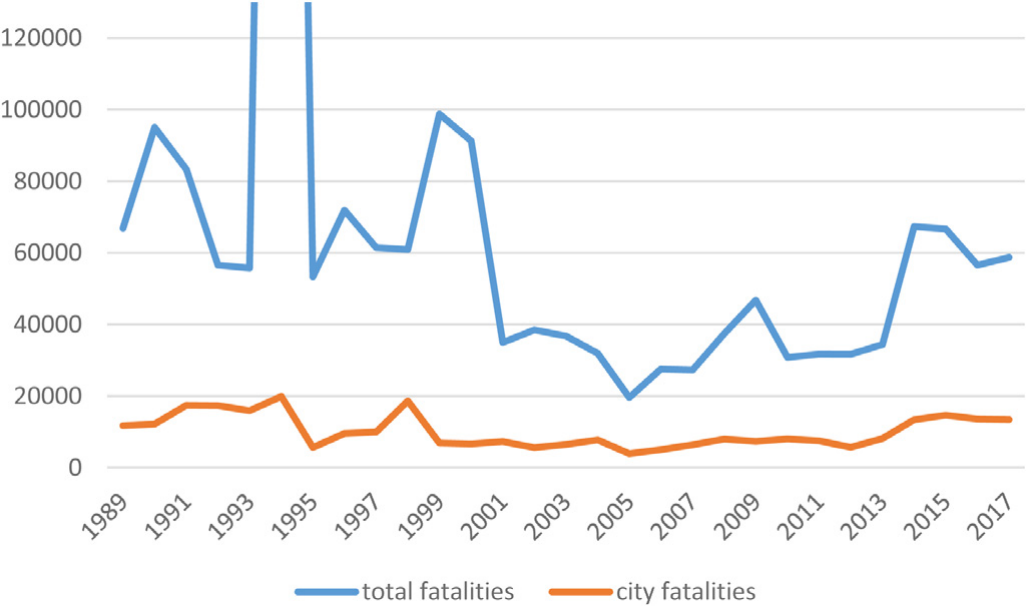
Patterns of violence (fatalities). Modified from Elfversson & Höglund [1].

Fig. 2 below presents the pattern over time in terms of fatalities. The blue line denotes the total number of fatalities from armed conflict each year, whereas the red line denotes the number of fatalities that were incurred in cities. The peak in total fatalities in 1994 (outside the graph area) captures the genocide in Rwanda. For cities (the red trend line), high levels of fatalities can be observed in the period up to 1994, during which for instance the war in Angola featured siege warfare in major cities; in 1998, when the wars in Afghanistan, Sudan and Republic of the Congo featured high levels of urban warfare; and from 2014 onwards, when for instance the war in Iraq heavily affected cities. Since 2014, around half of the fatalities in the war in Iraq have been incurred in the cities, including Mosul, Ramadi, Fallujah and Baghdad.

**Fig. 3:**
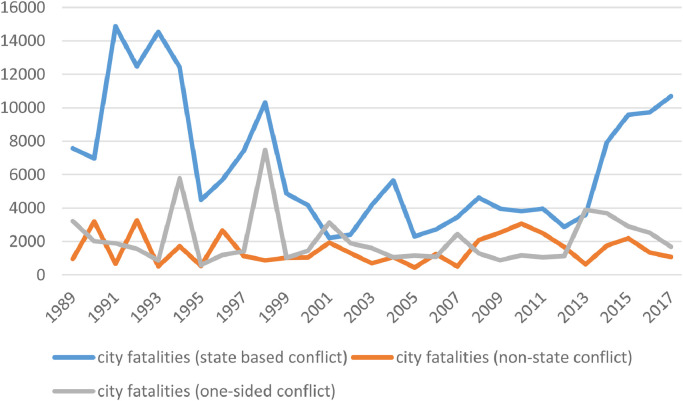
Patterns of violence (fatalities), state-based conflict.

The UCDP collects data for three categories of organized violence: state-based conflict, non-state conflict, and one-sided violence against civilians. The first category includes armed conflict between states, as well as armed conflict between a state and one or more rebel groups. For an in-depth analysis of urban-rural patterns in state-based conflict, see Elfversson & Höglund [1]. The second category includes fighting between rebel groups, militias, and communal groups. The third category includes events where organized actors (including the state) attack civilians. Fig. 3 below illustrates fatalities incurred in cities for each category, over time.

**Fig. 4:**
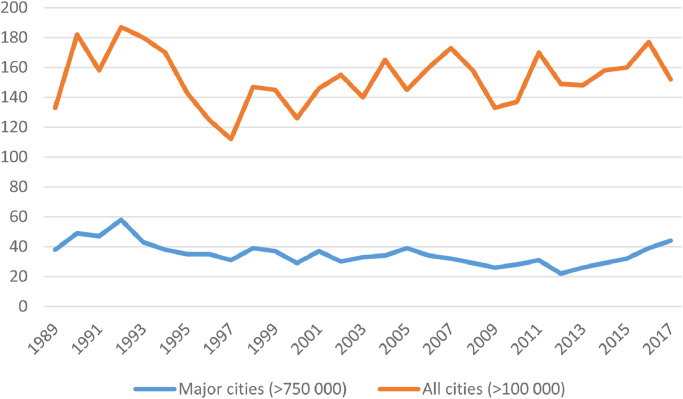
Number of cities affected by UCDP-GED violence over time.

**Fig. 5:**
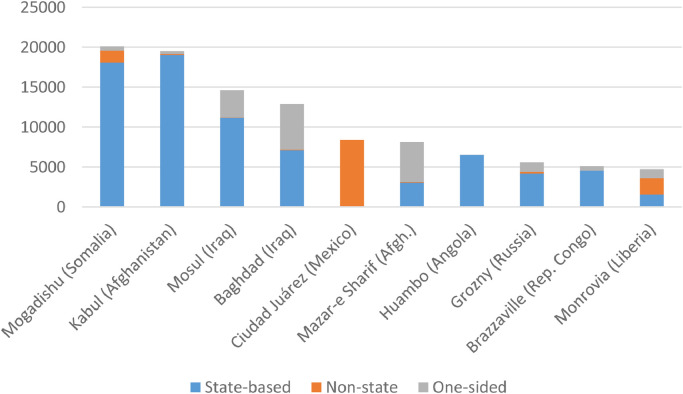
Most affected cities (excluding Rwanda and Syria), 1989-2017.

The data can also be used for comparison across different cities. Fig. 4 below shows the total number of cities affected by armed conflict over time (for all cities, as well as for major cities with at least 750 000 inhabitants). Fig. 5 illustrates the ten cities with the highest total number of fatalities during 1989-2017. Two countries are excluded: Syria, which is not included in UCDP GED v. 18.1, and Rwanda, due to high uncertainty in what share of fatalities during the genocide were incurred in Kigali (the bulk of this violence is coded as a summary event, spanning the entire country). Fig. 5 indicates that most of the worst affected cities are the scenes of state-based conflict. However, in Ciudad Juárez, all the recorded violence is non-state, primarily between rival gangs. In cases such as Baghdad and Mazar-e Sharif, a high proportion of the fatalities are made up of one-sided violence against civilians, such as massacres commited by both rebels and state forces.

## 2 Experimental Design, Materials and Methods

To identify armed conflict events taking place in cities, I matched the UCDP GED [2] (version 18.1) to data from the United Nations Statistics Division (UNSD) [3]. I relied on the UNSD data on city population, which are based on official demographic data from national statistical offices.

In the first step, I identified all countries with UCDP GED events, including states that have formed since 1989 from larger states which experienced UCDP GED events (e.g. the Baltic states, Montenegro). Next I extracted all cities in these countries from the UNSD data. I included only those cities that had at least 100 000 inhabitants in the city proper at some point since 1989. This numerical threshold was chosen because population centers of this size are expected to feature characteristics associated with cities: density, heterogeneity, high economic activity, and a certain political importance. Additionally, I included national capitals which did not meet this threshold (in effect, this concerns three cases: Port-of-Spain, Trinidad; Moroni, Comoros; and Honiara, Solomon Islands).

Given concerns about the reliability of city statistics – which include issues of politicized national censuses, poor infrastructure for gathering population statistics, and country variations in precisely how cities are delimited (see e.g. Jerven [4] and Satterthwaite [5]) – I took further steps to validate the information. For all countries where most recent data in the UN database is from 2000 or earlier, or where data quality is known to be low, I consulted alternative sources (for instance, this concerns Sierra Leone where the most recent entry in the UNSD data was from the 1980s and at that time only one city exceeded 100 000 inhabitants). For this triangulation, I relied on original national censuses where possible, as well as other data repositories (notably, Brinkhoff's City Population data [6]). In some cases, estimates were used (for instance, war-torn Somalia has not been able to conduct a proper census for a very long time, and we relied on UN estimates to expand the list of cities beyond those that had at least 100 000 citizens at the time of the last census).

The resulting list, which includes (for countries where UCDP events were recorded) all cities with a recorded (or in some cases, estimated) population of at least 100 000 at some point since 1989, contains a total 3956 cities.

In the next step, I matched the cities on the list to the UCDP GED (v 18.1). All the events contained in the UCDP GED were coded according to whether or not they occurred in one of the cities on my list, based on the location description in the dataset (“where”). In UCDP coding procedure, “where” is standardized so that all events taking place within a certain city are assigned to that city, rather than to a city sub-location (see Högbladh, [7]). This matching was conducted manually, taking into account cases where there are several alternative spellings for city names or where city names have changed over time. In cases with uncertainty, the event georeferences included in UCDP GED were used to confirm the match. Only events coded with high precision were assigned to cities. Summary events, and events with low geoprecision, are often assigned to a higher-order administrative unit in the UCDP data and coded “0” in my dataset. Meanwhile, events that occur in major cities are generally well reported and therefore tend to be coded with high precision.

In the CACE dataset, a dichotomous variable (CITY) indicates whether “where” matches a city according to my operationalization. Out of the total 3956 cities identified at the outset, 974 saw at least one event of organized violence as defined and coded by the UCDP. A separate dichotomous variable (CAPITAL) indicates if the event took place in a capital city. Furthermore, I include two additional dichotomous variables, MAJORCITY and TOP3CITIES which denote cities with a population of at least 750 000, and the three largest cities in each state, respectively.

Just as the underlying UCDP GED dataset, the CACE dataset covers the period 1989–2017, and is global in scope (except for Syria, which is not included in UCDP GED 18.1).

## Declaration of Competing Interest

The author declares that she has no known competing financial interests or personal relationships which have or could be perceived to have influenced the work reported in this article.

## Appendix Supplementary materials

Image, application 1

Image, application 2

Image, application 3
