# Supplementary material for: Cities and armed conflict: A systematic urban-rural coding of UCDP conflict events data
Source: Data Brief. 2021 Nov 7;39:107554. doi: 10.1016/j.dib.2021.107554 (PMC8603016; doi:10.1016/j.dib.2021.107554)
Supplement: Supplementary file 2 [file mmc2.pdf]

Codebook: Cities and Armed Conflict Events (CACE)

Emma Elfversson, Department of Peace and Conflict Research, Uppsala University

Version: 2020-10-30

If using this data, please cite: [*Elfversson, forthcoming*]

The Cities and Armed Conflict Events (CACE) dataset constitutes an extension of the Uppsala Conflict Data Program (UCDP) Georeferenced Events Dataset (GED) (Sundberg & Melander, 2013). The UCDP GED systematically codes events of violence for armed conflicts which result in at least 25 battle-related deaths (Sundberg & Melander, 2013). The Cities and Armed Conflict Events (CACE) dataset provides a systematic coding of whether these armed conflict events took place in cities. To identify which events of armed conflict took place in cities, the data was manually matched to data from the United Nations Statistics Division (UNSD, 2017).

IDENTIFYING CITIES

Source: United Nations Statistics Division

Data downloaded from:
http://data.un.org/Data.aspx?q=city+population&d=POP&f=tableCode%3a240 (2017-10-23)

From the UN cities data, all cities in countries with UCDP GED events (using UCDP GED v 18.1, covering 1989-2017) were extracted, making up a total list of 3956 cities. [1] Cities were only included if they had at least 100 000 inhabitants.[2] In addition, cities were included if they had less than 100 000 inhabitants but constituted the national capital.[3] For the population size, the "total population estimate" for the "city proper"[4] was used. Note that cities were included if they exceeded the population threshold at any time during time series according to available data; this was deemed important not least because some war-affected cities shrink as a result of being exposed to armed conflict – Jaffna in Sri Lanka is an example. A few states were not included in the UNSD data; for these, an alternative source (the

[1] Note that not all cities experienced UCDP-GED events. 974 of the 3956 cities experienced one or several UCDP-GED events.

[2] A note about the validity of this operationalization of "Cities". 100 000 means very different things in different contexts/countries (in USA may be the 2000th largest town, while in another case the largest) and does not capture other dimensions of what we think of as a city (cosmopolitanism, political centrality, etc). Alternatives considered was to find each country's own list of official cities – i.e. what is considered/defined as a city case by case – but this proved unfeasible (impossible to find for many cases) and in some cases includes very small towns, e.g. in Bosnia-Hercegovina towns with <20 000 inhabitants which also does not match our theoretical notion of a city.

[3] For capitals below 100 000, this is noted in the "comment" variable to allow filtering out. This only concerns Port-of-Spain (Trinidad), Moroni (Comoros) and Honiara (Solomon Islands).

[4] In some cases, there is only population data available for the larger "Urban agglomeration" and this was used instead: This applies fully or partially to Argentina, Bangladesh, Namibia, Belarus, Estonia, Laos and Guinea).

World Cities Database from Simplemaps which is based on US governmental sources) was used instead.[5]

To increase confidence that cities in line with this definition are correctly identified, a few additional steps were taken to triangulate the information. For all countries where the most recent data in the UNSD database was from 2000 or earlier, or where population data quality is known to be low, additional sources were consulted (for instance, for Sierra Leone the most recent data was from the 1980s and then only one city had over 100 000 inhabitants). For triangulation we relied on original national censuses where possible, as well as other data repositories (notably, Thomas Brinkhoff: City Population, http://www.citypopulation.de). In some cases, estimates were used (a few states such as Somalia have not been able to conduct a proper census for a very long time and we relied on UN estimates based on the most recent census and projected growth).

In summary, the first step identified (for countries affected by UCDP violence) all cities with a recorded (or in some cases, estimated) population of at least 100 000 at some point since 1989.

MATCHING EVENTS TO CITIES

In the next step, all events in the UCDP GED were coded based on whether or not they took place in a city on the list identified in the first step. This matching was conducted manually, taking into account that many city names have multiple spelling options and a few have changed name over time.

A separate dummy variable denotes whether the event takes place in *capital*.[6] The data is taken from the ICOW Historical State Names data set (http://www.paulhensel.org/icownames.html). Capitals are only counted as such once the country is independent (i.e. the capitals of the previous FYR and Soviet states; Dili (East Timor) only after the end of Indonesia's annexation).

Location information in UCDP-GED

For more information on these variables, see the UCDP GED codebook (Högbladh, 2020).

where_prec: "The precision with which the coordinates and location assigned to the event reflects the location of the actual event."
Comment: In CACE, only events with high precision (1 or 2) are assigned to cities. The rationale is that when an event takes place in a major city, the location is usually known – low geographic precision (e.g., an event reported as taking place "in xx province") is usually due to an event occurring in a remote area.

where_coordinates: "Name of the location to which the event is assigned."
Comment: This variable is used to match the events to the UN cities data.

[5] This concerns Angola, Afghanistan, Cambodia, DRC, Haiti, Libya, Morocco, Somalia, Sudan, Tunisia, Turkey, and United Arab Emirates.
[6] For countries with several (e.g. official/administrative), all are included (e.g. South Africa). The variable is allowed to vary over time e.g. Lagos 1989-1991 and Abuja December 1991 onwards in Nigeria.

Latitude: "Latitude (in decimal degrees)"
Longitude: "Longitude (in decimal degrees)"
<u>Comment:</u> The coordinates were used to double-check that city events were not missed, since all events taking place within a certain city have the same coordinates according to UCDP geocoding procedure (Högbladh, 2020, p. 19).

Cities that change state/country

FYR – checked all constitutive countries against full city list

Soviet union (1991) – Ukraine, Belarus, Armenia, Azerbadjan, Estonia, Georgia, Kazakhstan, Kyrgyzstan, Latvia, Lithuania, Moldova, Tajikistan, Turkmenistan, Uzbekistan (→ checked cities in all these states against events for Russia/Soviet Union)

Sudan/South Sudan (→ checked cities in both states against events for Sudan)

Indonesia/Timor Leste (→ checked cities in both states against events for Indonesia)

Comments on unclear/borderline cases

Airports: These are usually located outside city proper, but can be considered rather "urban" in many respects. City airports are coded 0 in CACE (unless they are coded with the city's coordinates in UCDP GED), but can easily be identified and recoded (by searching "airport" in "where_coordinates").

Several *low precision events* are coded with town/city coordinates; in CACE these are not coded as cities (based on reasoning above, i.e. if it happens in the city this is probably known), but a comment is included for these cases.

VARIABLES IN THE DATASET

In addition to the variables described below, the CACE dataset contains the full set of variables present in the UCDP GED v 18.1. For a description of these variables, see the UCDP GED codebook (Högbladh, 2020). The CACE variables are clearly marked in the dataset (gray background).

<u>City</u>: A dichotomous variable which takes the value 1 if "where_coordinates" is a city which had at least 100 000 inhabitants at some point since 1989 (see "Matching events to cities" above).

<u>CACE cityname</u>: The name of the city in the CACE data (usually the same as in UCDP GED, but without the suffix "city" or "town")

<u>Capital</u>: A dichotomous variable which takes the value 1 if "where_coordinates" is a national capital.

Majorcity: A dichotomous variable which takes the value 1 if “where_coordinates” is a city which had at least 750 000 inhabitants. Note: this is coded based on the identified population value that is recorded closest to the middle of the time series.

Top3cities: A dichotomous variable which takes the value 1 if “where_coordinates” is a city that is among the three largest cities in the country.

Comment: A text comment on the coding of the previous variables, if necessary. In a few rare cases the coding was ambiguous dues to the location name, summary nature of an event, or the coordinates provided and a comment on the coding decision is warranted.

REFERENCES

Högbladh, S. (2020). *UCDP GED Codebook version 20.1*. Retrieved from https://ucdp.uu.se/downloads/ (accessed 2020-10-30).

Sundberg, R., & Melander, E. (2013). Introducing the UCDP Georeferenced Event Dataset. *Journal of Peace Research, 50*(4), 523-532. doi:10.1177/0022343313484347

UNSD. (2017). *City Population by Sex, City and City Type*. New York: United Nations StatisticsDivision. Retrieved from http://data.un.org/Data.aspx?q=city+population&d=POP&f=tableCode%3a240 (2017-10-23)
